# Reduction of gastrointestinal tract colonization by *Klebsiella quasipneumoniae* using antimicrobial protein KvarIa

**DOI:** 10.1186/s13099-022-00492-2

**Published:** 2022-04-26

**Authors:** Indre Karaliute, Rima Ramonaite, Jurga Bernatoniene, Vilma Petrikaite, Audrius Misiunas, Erna Denkovskiene, Ausra Razanskiene, Yuri Gleba, Juozas Kupcinskas, Jurgita Skieceviciene

**Affiliations:** 1 Institute for Digestive Research, Laboratory of Clinical and Molecular Gastroenterology, Lithuanian University of Health Sciences, Mickeviciaus st. 9, 44307 Kaunas, Lithuania; 2 Department of Drug Technology and Social Pharmacy, Lithuanian University of Health Sciences, Sukileliu Pr. 13, 50161 Kaunas, Lithuania; 3 Laboratory of Drug Targets Histopathology, Institute of Cardiology, Lithuanian University of Health Sciences, 50162 Kaunas, Lithuania; 4 Nomads UAB, Geležinio vilko 29A, 01112 Vilnius, Lithuania; 5 Nomad Bioscience GmbH, Biozentrum Halle, Weinbergweg 22, 06120 Halle (Saale), Germany; 6 Department of Gastroenterology, Lithuanian University of Health Sciences, 44307 Kaunas, Lithuania

**Keywords:** *Klebsiella quasipneumoniae*, Klebicins, KvarIa, Haemolysin gene, Bacteriocins

## Abstract

**Background:**

*Klebsiella quasipneumoniae* is an opportunistic pathogen causing antibiotic-resistant infections of the gastrointestinal tract in many clinical cases. Orally delivered bioactive *Klebsiella*-specific antimicrobial proteins, klebicins, could be a promising method to eradicate *Klebsiella* species infecting the gut.

**Methods:**

Mouse infection model was established based on infection of antibiotic-treated BALB/C mice with *K. quasipneumoniae* strain DSM28212. Four study groups were used (3 animals/group) to test the antimicrobial efficacy of orally delivered klebicin KvarIa: vehicle-only group (control, phosphate-buffered saline), and other three groups with bacteria, antibiotic therapy and 100 µg of uncoated Kvarla, 100 µg coated KvarIa, 1000 µg coated-KvarIa. Because of the general sensitivity of bacteriocins to gastroduodenal proteases, Kvarla doses were coated with Eudragit®, a GMP-certified formulation agent that releases the protein at certain pH. The coating treatment was selected based on measurements of mouse GI tract pH. The quantity of *Klebsiella* haemolysin gene (*khe*) in faecal samples of the study animals was used to quantify the presence of *Klebsiella*.

**Results:**

GI colonization of *K. quasipneumoniae* was achieved only in the antibiotic-treated mice groups. Significant changes in *khe* marker quantification were found after the use of Eudragit® S100 formulated klebicin KvarIa, at both doses, with a significant reduction of *K. quasipneumoniae* colonization compared to the vehicle-only control group.

**Conclusions:**

Mouse GI tract colonization with *K. quasipneumoniae* can be achieved if natural gut microbiota is suppressed by prior antibiotic treatment. The study demonstrates that GI infection caused by *K. quasipneumoniae* can be significantly reduced using Eudragit®-protected klebicin KvarIa.

## Background

*Klebsiella* is a gram-negative and facultative anaerobic bacterium of the *Enterobacteriaceae* family that colonizes various environmental niches including normal flora of the human mouth or intestines [1, 2]. Pathogenic *Klebsiella* species, such as *K. pneumoniae* and *K. oxytoca*, are the most prevalent infections acquired in hospital (HAI) [2, 3]. Recent studies have identified *K. quasipneumoniae* as a new species distinguishable from *K. pneumoniae. K. quasipneumoniae* has been shown to act as an etiological agent in a number of clinical *Klebsiella*-related infection cases, but has often been misidentified as *K. pneumoniae* in HAI [4, 5]. *K. quasipneumoniae* colonizes the intestinal tract, which can lead to virulent urinary tract and abdominal infections [3–6]. Furthermore, this type of bacteria shows high rates of resistance against antibiotics, and some strains have been characterized as pan-drug resistant, making these infection extremely difficult to treat [7, 8]. For this reason, the development of new antimicrobial agents is required to mitigate bacterial infections, typically acquired by patients while in-hospital.

Colicin-like bacteriocins, produced by gram-negative bacteria, may be a potential alternative to traditional antibiotics [9, 10]. Colicin-like bacteriocins are a heterogeneous family of proteinaceous toxins, which are capable of killing closely related bacteria, those belonging to the same species or, sometimes, the same genus [11]. This property makes them attractive as therapeutics since they offer a more targeted approach than conventional antibiotics. Indeed, one of the major issues with conventional antibiotics is the dysbiosis induced by the broad-range killing of bacteria [12, 13]. Most importantly, the mechanisms of bacterial killing by bacteriocins are fundamentally different from those by antibiotics. Consequently, they are active against multi-drug and pan-drug resistant pathogens.

The authors have previously identified and characterized several *Klebsiella* colicin-type bacteriocins, klebicins, which exhibit significant and broad activity against the pathogenic *Klebsiella* species. Orally delivered klebicins have a potential as an excellent means to eradicate intestinal infections in hospitalized patients that are caused by the multidrug-resistant *Klebsiella* strains. However, the proteinaceous nature of bacteriocins makes them susceptible to quick inactivation by gastroduodenal enzymes. Therefore, for the oral administration of klebicins, they must be encapsulated or formulated for gastroduodenal protection, for the release in the small and large intestine.

In this study, the possibility to use klebicins to eradicate intestine colonizing Klebsiella was tested in K. quasipneumoniae–KvarIa model. Klebicin KvarIa is pore forming bacteriocin, highly active against K. quasipneumoniae [14]. We developed a mouse model of *K. quasipneumoniae* intestinal colonization, tested the pH condition of the mouse GI tract, and established a suitable coating for klebicin KvarIa. We also evaluated the antimicrobial activity of the orally delivered Eudragit S100-formulated klebicin in the mouse intestinal tract. We confirmed that, even without further bacteriocin engineering and improvement, bacteriocins could be employed as oral antimicrobials for efficient control of antibiotic-resistant *Klebsiella*.

## Methods

### Aim of this study

To investigate the antimicrobial effectiveness of the klebicin KvarIa in a mouse model of *K. quasipneumoniae* gastrointestinal (GI) colonization.

### Mouse models

Two different animal study designs were used for *K. quasipneumoniae* colonization and KvarIa treatment. For both studies, 8–10 weeks old, BALB/c strain, female (n = 8; 19–25 g) and male (n = 16; 22–27 g) mice were purchased from the Lithuanian University of Health Sciences vivarium of laboratory animals. All regulated procedures on living animals were approved by The Lithuanian Ethics Committee of Biomedical Research (Protocol no. G2-119).

### GI model of *K. quasipneumoniae* (DSM28212) colonization and KvarIa therapy

**Fig. 1:**
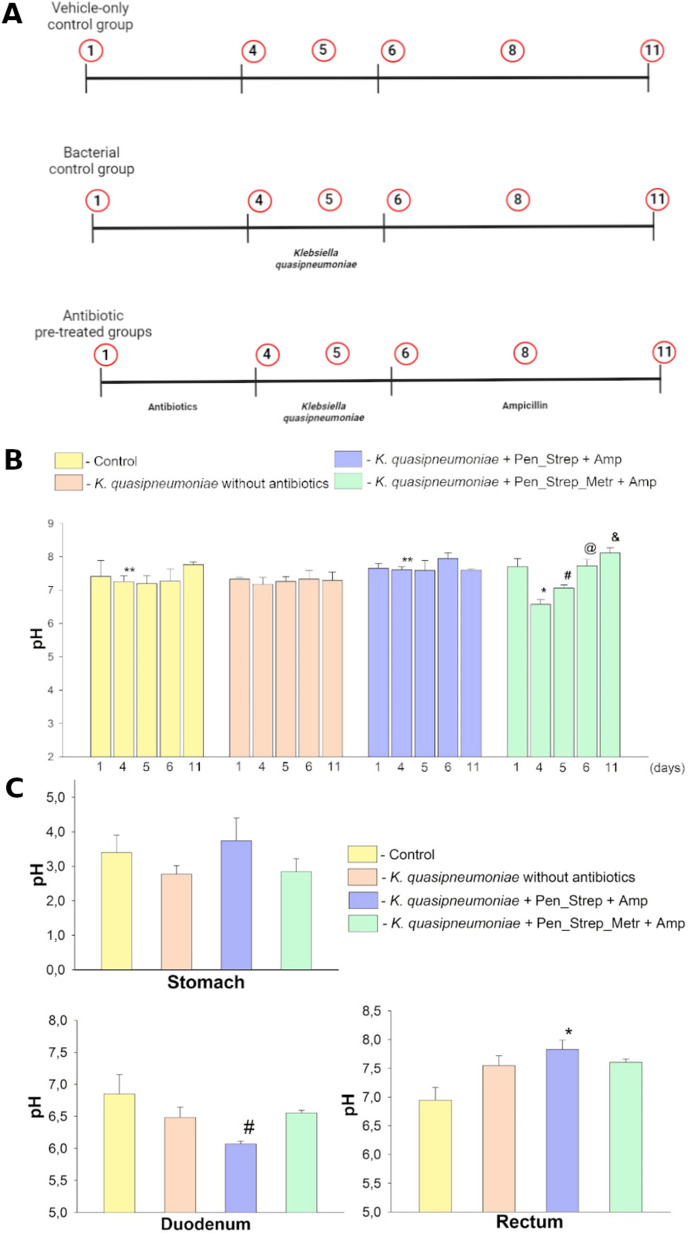
Experimental design of *K. quasipneumoniae* colonization and pH of GI. **A** The design of the study of mice gut colonization by K. *quasipneumoniae*. 1st Vehicle-only control group with natural microbiota (n = 3); 2nd Bacterial control group (n = 3); 3rd, 4th antibiotic pre-treated groups [(i) penicillin, streptomycin (pen_strep group) or (ii) penicillin, streptomycin, and metronidazole (pen_strep_met group)]. Samples were collected on circled days. **B** The pH values measured in the samples of rectum excreta throughout the layout of the experiment protocol in order to determine the changes in the GI tract using different substances*.* The control group showed an average of 7.34 pH with the lowest being 6.99 pH and the highest 7.76 pH; the infected mice group without antibiotics showed almost no changes in pH measures through the days (av. 7.27 pH); *K. quasipneumoniae* infected group was treated with antibiotic therapy (penicillin, streptomycin) and showed stable results (av. 7.7 pH) with an exception on the 6^th^ day of the experiment (7.94 pH); the last group, which was infected with *K. quasipneumoniae* showed the most noticeable changes during the combined antibiotic (penicillin, streptomycin, metronidazole) treatment and bacterial colonization with the average of 7.44 pH fluctuating from 6.57 pH to 8.11 pH. No significant differences were found in or between the groups. **C** Overall differences in values of stomach, duodenum, and rectum of pH measurements between groups. The lowest pH was seen in the stomach (2.34 – 5.46; avg. 3.3). The pH of the small intestine varied between 5.97 and 6.77 (avg. 6.5), with the large intestine showing the highest pH (6.74–8.15; avg. 7.4). A significant difference (p < 0.05) in pH measures was found in the duodenum between 3rd and 4th groups (#), as well as between 1st group and 3rd (*) in rectum

**Fig. 2:**
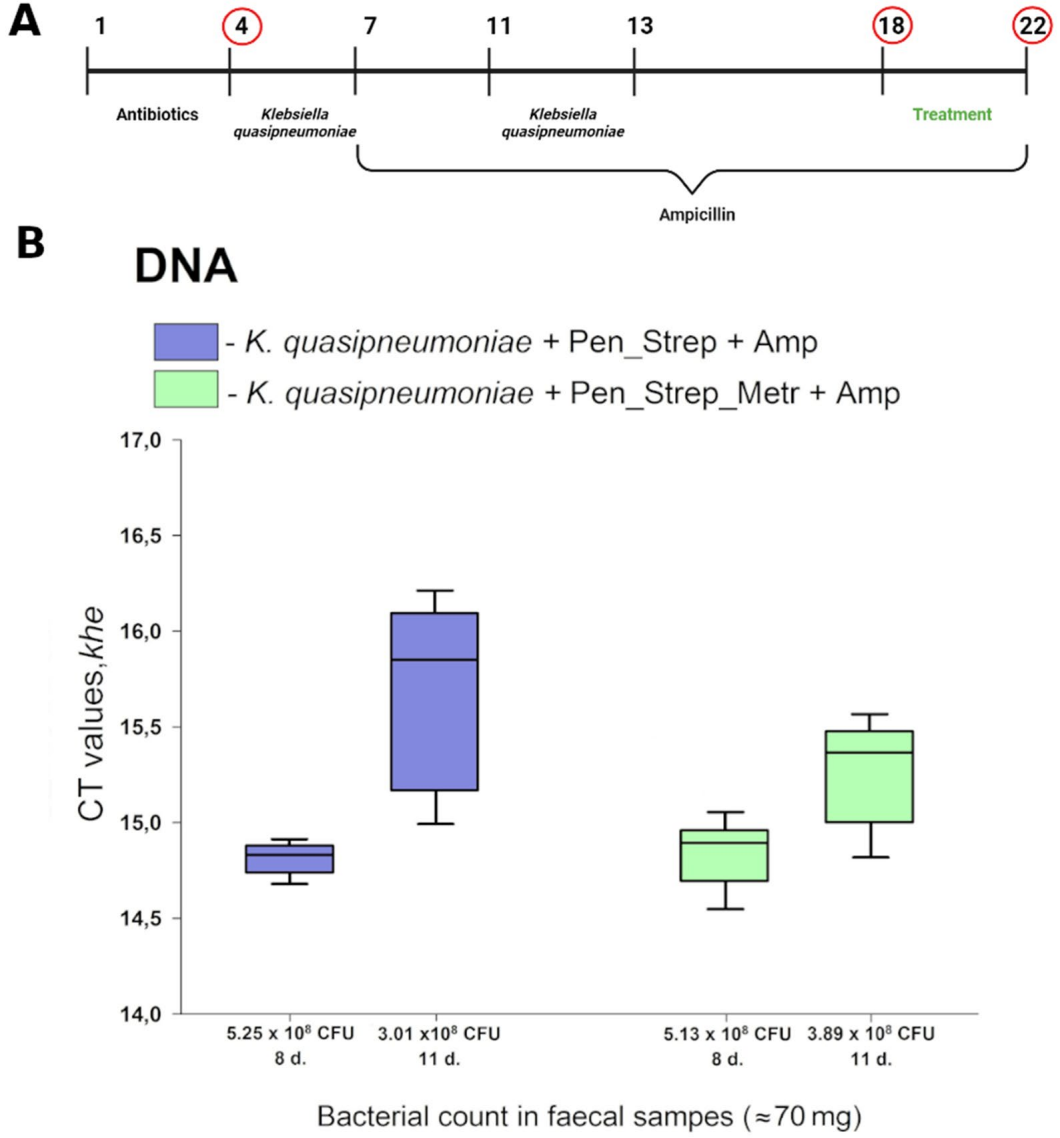
Experimental design of *K. quasipneumoniae* therapy and assessment of *Klebsiella quasipneumoniae* colonization. **A** The design of the study of mice gut colonization by *K. quasipneumoniae* and KvarIa treatment. All four experimental groups had antibiotic pretreatment, ampicillin therapy, bacterial administration, and different treatment for infection (PBS; uncoated-KvarIa; Eudragit S100-coated KvarIa 100 µg; Eudragit S100-coated KvarIa 1000 µg). Samples were collected on circled days. **B** Amplification of *khe* gene in mice faeces after the introduction of *K. quasipneumoniae. khe* gene was detected by RT-PCR. A lower CT value indicates a higher number of bacteria. The 4th day’s samples were used as a control group because they did not show any bacterial colonization. Statistically significant differences between mice groups (K. quasipneumoniae + Pen_ Strep + Amp *vs K. quasipneumoniae* + Pen_ Strep_Met + Amp) were not determined. Pen–penicillin, Strep–streptomycin, Met–metronidazole, Amp–ampicillin

*Klebsiella quasipneumoniae* clinical isolate DSM28212 was used for GI tract colonization in four different study groups containing three mice per group (m = 2; f = 1). Vehicle–only control group was monitored for any changes in the natural host-microbiota without any additional procedures during the period of the experiment. The ability of *K. quasipneumoniae* to colonize the GI tract without antibiotic pre-treatment to disrupt the host-microbiota was tested. In order to mimic hospital-acquired infections two groups were given different combinations of antibiotic treatment before infection (penicillin (2000 U/ml) + streptomycin (2 mg/ml), (pen_strep); penicillin (2000 U/ml) + streptomycin (2 mg/ml) + metronidazole (1 g/L) (pen_strep_met)) (study design in Fig. 1A). These particular antibiotics were chosen because of their broad mechanism of action against gram-negative and gram-positive bacteria. For KvarIa therapy testing the same composition of antibiotic pre-treatment was used as in the previously described study. Three groups (**A; B; C**) received 10^9^ cfu of *K. quasipneumoniae* orally by pipette feeding once per day. Afterward, ampicillin (500 mg/l) therapy was used to maintain as low as possible viability of other than *K. quasipneumoniae* bacteria. From day 18th group A was given 100 µg of uncoated KvarIa, groups B and C were given 100 µg and 1000 µg of Eudragit S100-coated KvarIa respectively (detailed study design Fig. 2A). For each mouse, faecal pellets were sampled.

### Determination of pH of the gastrointestinal tract

As shown in Fig. 1A, the samples of rectum excreta were collected on six different days during the experiment. Acquired samples were homogenized with deionized water (1:10 ratio) and pH was determined using pH METER Mettler Toledo (Belgium) with the Inlab Ultra-Micro electrode. In addition, the pH was measured in the samples taken from the GI tract during the laparotomy dissection (the intestinal tract was divided into three sections: the stomach, the duodenum, and the rectum).

### Klebicin production in plants and purification

*Klebsiella* bacteriocin KvarIa was expressed in *Nicotiana benthamiana* transient expression system and purified as previously described in Denkovskiene et al. [14].

### Coating of KvarIa

5% Eudragit S100 solution was prepared by dissolving 0.5 g Eudragit S100 (Evonik Industries, Germany) in 10 ml of miliQ H_2_O and by sonication in an ultrasonic bath for 30 min at 25 °C. 250 µg of KvarIa was dissolved in 200 µg of 5% Eudragit S100. The obtained solution was lyophilized at − 51 °C for 24 h.

### Simulated gastric digestion and residual KvarIa activity evaluation by soft agar radial diffusion assay

Protein samples (KvarIa and Eudragit S100-coated KvarIa) were dissolved in simulated gastric buffer (0.15 M NaCl, pH 2), at a concentration of 1 mg/ml and incubated at 37 °C with rotation at 200 rpm for 10 min. 0.025 mg (80–113 U) of pepsin from porcine gastric mucosa was added to 1 mg of protein (pepsin:protein ratio 1:40). Aliquots of reaction (50 µl) were removed at different time points (0.5 min, 5 min, 10 min, 20 min, 30 min, and 60 min after the addition of the pepsin). Digestions were stopped by raising the pH to 6.5 by the addition of 0.5 M ammonium bicarbonate to inactivate pepsin. The pH of samples was adjusted to 8.0 to get Eudragit S100 coat dissolved. The dilutions of all samples by ratio 1:2 were made in distilled water and 5 µL drops of diluted samples were applied on *K.quasipneumoniae* DSM28212 MHA plates for soft agar overlay assay.

Soft-agar overlay assays were performed as described by Denkovskiene et al. [14], with some modifications. *K. quasipneumoniae* DSM28212 overnight culture was equalized to OD_595_ = 1.0 in Muller-Hinton medium and diluted 100-fold in 0.8% (w/v) top agar preheated in a 55 °C water bath. Mixed overlay components were poured on plates containing solid medium (Muller-Hinton containing 1.5% (w/v) agar). Sterile Whatman paper discs (6 mm diameter) were placed on the surface of the soft-agar medium containing bacterial test strain and 5 µl of protein dilutions were applied to the discs. The plates were incubated overnight at 37 °C and the diameter of klebicin inhibition zones was measured.

Briefly, plant-produced KvarIa and Eudragit S100-coated KvarIa were mixed with SGF at the recommended concentration and incubated for up to 60 min, sampling every few minutes and assessing the digestion of the protein into fragments by SDS-PAGE. Coomassie staining on gels was used to visualize protein decomposition and estimate the MW of peptide products. This method was only used for uncoated KvarIa, as Eudragit S100 distorted protein migration on the SDS-PAGE gel.

### Nucleic acid extraction and synthesis of the cDNA

Bacterial DNA and RNA from rectum excrement samples were extracted using the AllPrep PowerFecal DNA/RNA Kit. Up to 100 mg of faeces sample were used for the extraction procedures. The quantity and quality of extracted nucleic acids were evaluated by NanoDrop 2000 (Nanodrop Technologies, Wilmington, DE, USA). Subsequently, cDNA was synthesized using a High-Capacity cDNA Reverse Transcription Kit (Thermo Fisher Scientific, Lithuania). 18 ng of cDNA was added into the qualitative real-time PCR (qRT-PCR) reaction. All processes were completed upon the manufacturer’s instructions.

### Quantitative assessment of *Klebsiella quasipneumoniae* using Real-Time–PCR

The haemolysin gene (*khe*) was chosen as the qualitative marker for *Klebsiella* identification [15, 16]. The standard curve was created based on DNA samples of *K. quasipneumoniae* (DSM28212) to test the generated primers’ efficiency. DNA-based standard curve. 10^3^, 10^5^, 10^6^, 10^8^, 10^9^, and 10^10^ CFU of *K. quasipneumoniae* in 200 µl were subjected to DNA extraction with QIAamp Fast DNA Stool Mini Kit (protocol for liquid sample).

During this step, the reaction for the qRT-PCR was performed using TaqMan Universal Master Mix II with UNG, TaqMan probe (5-6FAM-CGCGAACTGGAAGGGCCCG-TAMRA-3), and primers (Forward: 5 -GATGAAACGACCTGA TTGCATTC-3, Reverse: 5 -CCGGGCTGTCGGGATAAG-3 (Applied Biosystems, JAV) following the manufacturer’s recommendations. The amplification of the *khe* gene was determined by ABI Fast 7500 System (Life Technologies, Carlsbad, CA, USA) according to standard protocol. Positive controls for DNA and RNA were isolated from *K. quasipneumoniae* and negative—isolated from *Esherichia coli*.

### Statistical analysis

The data were analysed using nonparametric tests. The difference between the four protocols groups throughout the layout of the experiment were analysed using Student’s independent *t*-test. Independent analyses were carried out using SPSS Version 19.0 and MiniTab 20.1.2 software packages. Results were considered statistically significant when *p* < 0.05 with ± 95% confidence intervals.

## Results

### Selection of bacteriocin Kvarla coating by pH measurements along the GI tract

In order to determine the efficient coating and delivery of the klebicins in the GI tract, firstly we performed the pH measurements in faeces and along the GI tract. The pH of the faecal samples is shown in Fig. 1B. The lowest pH was seen after penicillin, streptomycin, and metronidazole treatment. Nonetheless, there was no observation of any statistically significant changes in GI tract pH between groups (average mean ± SD: vehicle-only control group 7.3 ± 0.52; bacterial control group 7.5 ± 0.74; pen_strep group 7.7 ± 0.29; pen_strep_met group 7.4 ± 0.62). The pH levels of the different GI tract sections was also measured after the mouse decapitation (the stomach 3.3 ± 0.92, small intestine 6.5 ± 0.34, the large intestine 7.4 ± 0.42; Fig. 1C) The significant differences in pH measures were found between the vehicle-only control group and pen_strep group (p = 0.004) in duodenum, vehicle-only control group and pen_strep_met group (p = 0.04) in rectum.

### Colonization of mice gut by *K. quasipneumoniae* is achieved only after disruption of natural microflora

The GI tract infection/colonization model was established in mice using *Klebsiella quasipneumoniae.* It was designed to reflect bacterial colonization in the host after the disruption of natural microflora with antibiotics therapy. There was no colonization of *K. quasipneumoniae* observed in the vehicle-only control group. The use of antibiotic pre-treatment ((i) penicillin, streptomycin or (ii) penicillin, streptomycin, and metronidazole), in order to disrupt the host microbiota, resulted in introduction of *K. quasipneumoniae* (4th day no bacterial counts were found (0 CFU/70 mg); (i) 8th day—5.25 × 10^8^ CFU/70 mg, 11th day—3.01 × 10^8^ CFU/70 mg and (ii) 8th day—5.13 × 10^8^ CFU/70 mg and 11th day—3.89 × 10^8^ CFU/70 mg) (Fig. 2). Our data showed that colonization of mice gut by *K. quasipneumoniae* can be established after eradication of natural gut microflora.

### Eudragit S100-coated KvarIa is partially protected from digestion by simulated gastric fluid

To find out if Eudragit S100-coated KvarIa is resistant to pepsin digestion, a simulated gastric digestion experiment was performed. Exposures of the proteins to Simulated Gastric Fluid (SGF, commercial acidic pepsin extract) were done using low enzyme-to-substrate ratios in order to increase the stringency and relevance of the digestibility assays. Methods were derived from [17–19].

**Fig. 3:**
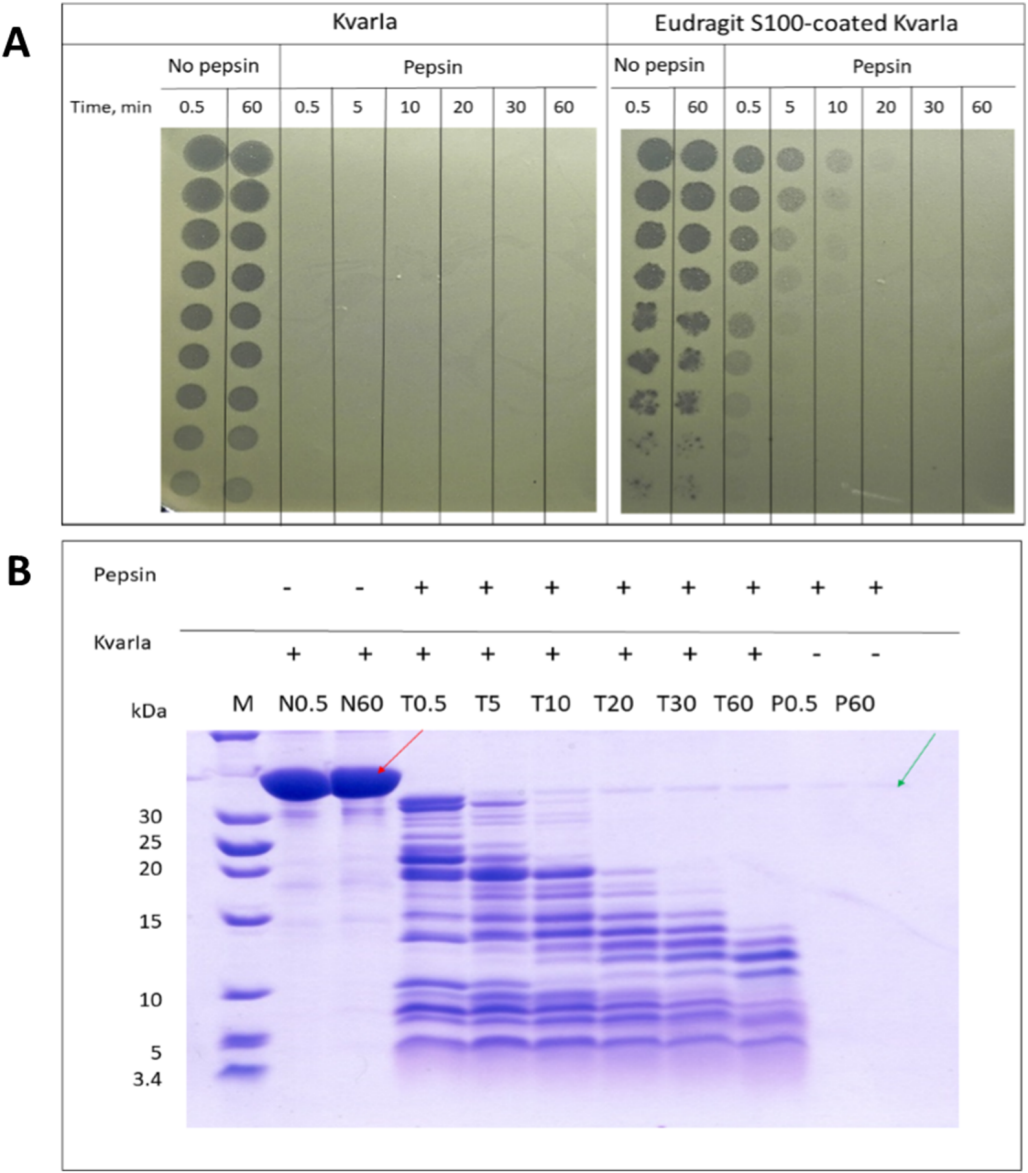
Evaluation of stability and activity of KvarIa after in vitro gastric digestion assay. **A** Evaluation of residual activity by agar diffusion assay. Protein samples were incubated at 37 °C, 200 rpm in gastric digestion buffer (pepsin:protein ratio 1:40). Aliquots of reaction (50 µl) were removed at different time points (0.5, 5, 10, 20, 30, and 60 min) and digestion was stopped by the addition of 0.5 M ammonium bicarbonate to inactivate pepsin. The pH of samples with coated KvarIa was adjusted to 8 to get Eudragit coat dissolved. The dilutions of all samples by ratio 1:2 were made in distilled water and 5 µL drops of diluted samples were applied on *K.quasipneumoniae* DSM28212 MHA plates for soft agar overlay assay. **B** SDS-PAGE of KvarIa after simulated gastric digestion. The 16% TRIS-Tricine gels; unstained marker—PageRuler™ Unstained Low Range Protein Ladder (ThermoFisher), 10 µl of each sample loaded per line. Pepsin appears as the stable band between 30 and 42 kDa markers (green arrow). The red arrow indicates the full-size KvarIa. Exposure of KvarIa to pepsin (1:40 pepsin:lysin wt:wt) in acidic SGF results in the rapid breakdown of the protein to lower MW degradation products. Sampling times are shown in minutes

It appears, that in conditions used (pepsin:protein ratio 1:40), protein coating with Eudragit S100 is able to provide temporal resistance to pepsin digestion. Coated KvarIa demonstrated still detectable activity in agar diffusion assay after 20 min of in vitro gastric digestion, while uncoated KvarIa was inactivated in simulated gastric juice very quickly, and completely lost its activity already after 0.5 min of digestion (Fig. 3). From the SDS-PAGE profile of uncoated KvarIa digestion products, it is apparent that uncoated klebicin in digested by pepsin very rapidly.

### Eudragit S100-coated KvarIa efficiently reduces *K. quasipneumoniae* amount in colon

**Fig. 4:**
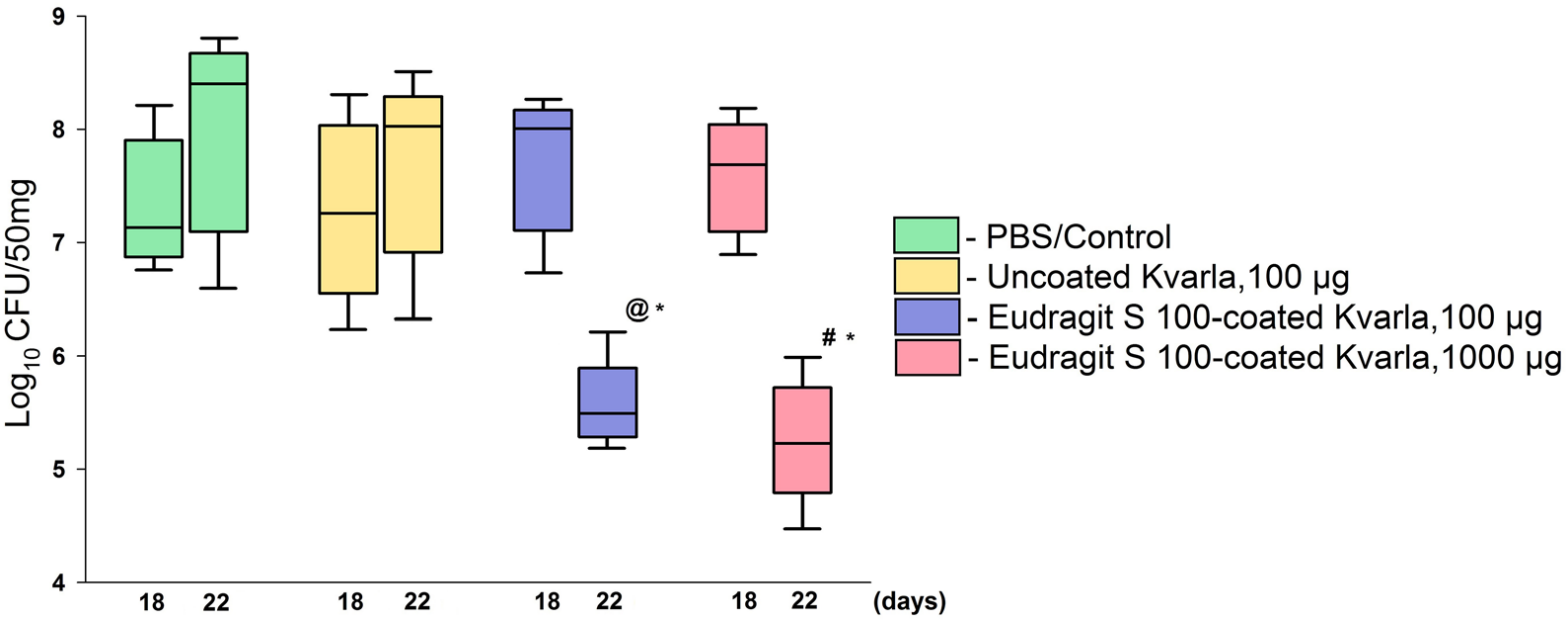
Bacterial counts in GI tract after KvarIa therapy. *khe* gene from DNA templates was determined using RT-PCR. A lower CT value indicates a higher number of bacteria (see Standard Curve). *khe* has not been detected in all study groups on the 4th day. * Statistically significant differences between 18th and 22nd days in all the experimental groups treated by Eudragit S100-coated KvarIa, p < 0.05. ^@^ PBS (control) and uncoated-KvarIa (100 µg) treated mice *vs* Eudragit S100-coated KvarIa (100 µg) treated group on the 22nd day, p < 0.05. ^#^ PBS (control) and Uncoated KvarIa (100 µg) treated mice *vs* Eudragit S100-coated KvarIa (1000 µg) treated group on the 22nd day, p < 0.05

We evaluated the effectiveness of Eudragit S100-coated KvarIa (100 µg; 1000 µg) in *K. quasipneumoniae* infection model. Three different combinations of recombinant klebicin were used: uncoated-KvarIa, Eudragit S100-coated KvarIa 100 µg, and Eudragit S100-coated KvarIa 1000 µg. The amplification of the *khe* marker gene was significantly higher in both Eudragit S100-coated KvarIa-treated mice groups than in the control (PBS) and uncoated-KvarIa-treated mice on the last day of the experiment (22nd day), meaning that bacterial counts were lower. As shown in Fig. 4 the bacterial counts were significantly lower after the treatment with Eudragit S100-coated KvarIa 100 µg and 1000 µg in contrast with the samples taken on the first day of bacteriocin administration (18th day). The amounts of *K. quasipneumoniae* changed from 6.3 × 10^7^ CFU/50 mg on the 18th day to 3.9 × 10^5^ CFU/50 mg on the 22nd day (p = 0.01) in the Eudragit S100-coated KvarIa 100 µg group and from 4.0 × 10^7^ CFU/50 mg on the 18th day to 1.6 × 10^5^ CFU/50 mg on 22nd day (p = 0.009) in the Eudragit S100-coated KvarIa 1000 µg group. No significant changes in bacterial counts were seen in the vehicle-only control group (PBS) and after the administration of uncoated-KvarIa.

## Discussion

A rapidly increasing number of antibiotic-resistant and/or highly virulent bacterial strains is a serious challenge faced by today’s healthcare system worldwide. Recent studies indicate that patients, with hospital-acquired multidrug-resistant *K. pneumoniae* infection, have a significantly higher risk of developing a subsequent infection caused by identical bacteria [20–23]. *K. quasipneumoniae* were initially thought to be asymptomatic carriage isolates until more recent reports highlighted their potential virulence and increased drug resistance [20–23].

*K. pneumoniae* has been extensively studied in many different animal models, including models for bloodstream infections, pneumonia, liver abscess, digestive and urinary tract infections [24, 25]. On the other hand, little is known about closely related species recently separated from *K. pneumoniae* such as *K. variicola* and *K. quasipneumoniae*. There were only limited animal studies with *K. variicola* such as experiments on the bacteria’s ability to colonize the intestinal tract and the host immune system response against this opportunistic pathogen [26, 27]. *K. quasipneumoniae* has been detected in the clinical settings during hospital infections, however, the species has not been tested in animal efficacy models, and mechanisms of infection by this bacterium are poorly understood. Therefore, the first goal of our study was to establish an animal model of *K. quasipneumoniae* intestinal colonization, in particular, identify the conditions that allow bacteria to successfully grow in the mouse intestinal tract. We demonstrate here that for successful colonization of mice gut by *K. quasipneumoniae*, the disturbance of natural gut microflora using antibiotic pre-treatment is necessary and sufficient.

Bacteria were not detected in the bacterial control group without prior antibiotic treatment (judged by *khe* amplification). Similar findings were observed in other *Klebsiella* mouse models where amoxicillin disruption of the gut microbiota was accompanied required for gut colonization and an enhancement of the virulence of *K. variicola* [27]. Other studies illustrated that mouse models of *K. pneumoniae* and treatment with antibiotics led to changes in the host microbiota and the development of a transient super-shedder phenotype, which displays the enhanced transmission efficiency of bacteria in the GI tract [28, 29]. Allegedly, the natural host microbiota activates the defence mechanisms against *K. quasipneumoniae* and inhibits colonization, whereas reduced microbial diversity might promote the ability to infect. However, the exact mechanisms causing *K. quasipneumoniae* colonization needs further investigation.

It is known that bacteriocins have antimicrobial activities against pathogenic microorganisms [30, 31]. Previous studies have identified various classes of bacteriocins (e.g.: colicin-like bacteriocins, tailocins, peptide microcins) and their potential applications in food technology, treatments of infection, and cancer [32–34]. Earlier, we demonstrated the antibacterial efficacy of *Klebsiella* bacteriocins, klebicins, in vitro using clinical *Klebsiella* isolates*.* Recombinant pore-forming bacteriocin KvarIa was identified as one of the most active klebicins; it showed the highest activity against *K. quasipneumoniae* strains and was also tested in vivo in a non-mammal animal model*, Galleria mellonella* larvae, demonstrating significant antibacterial effect [14]. In this study, we developed a mouse model of intestinal tract infection using *K. quasipneumoniae* and evaluated the effectiveness of Eudragit S100-coated KvarIa treatment with the main purpose of investigating the potential of klebicins as a clinical antimicrobials. The obtained results can further be translated to *K. pneumoniae* and other more clinically relevant *Klebsiella* species.

The authors determined the most effective coating for bacteriocin needed for delivery of the highest concentrations of the klebicin to the large colon. The pH in the GI tract is a substantial factor, affecting the solubility and stability of the drug and absorption through the intestinal tract mucosa. It can vary depending on the diet type, fed or fasted states, drugs, microbiota diversity, stress, and daily fluid intake. Henceforth, unsuitable pH causes the precipitation of drugs from the solution or the degradation of labile compounds [35–37]. Correspondingly, an assessment of pH levels in the GI tract was included in our study. We distinguished the increased pH level of the rectum content sample in the *K. quasipneumoniae* colonized mice groups treated with antibiotics. However, mice without antibiotics did not show any change in pH levels. Similar results were obtained by Shimizu and colleagues in ICR mice housed obtaining specific pathogen-free conditions, there the pH of the cecum and colon increased exceedingly in the experimental groups treated with antibiotics [38]. Therefore, the pH measurements of the GI tract were taken into account when choosing the most effective coating for KvarIa delivery.

In this study recombinant bacteriocins KvarIa ability to eliminate the intestinal tract infection was judged using *khe* gene quantification. We identified that both concentrations (100 µg and 1000 µg) of coated-KvarIa significantly reduced the infection in the GI tract of mice models. However, in our study, we did not achieve full eradication of the *K. quasipneumoniae.* KvarIa was encapsulated with Eudragit S100 releasing klebicin at pH above 7 and administered by oral gavage to infected (*K. quasipneumoniae*) mice. Debatably, klebicin activity could be suppressed or significantly lowered because of the gut microflora disruption or not full eradication, as well as, dependence on the pH level, which can fluctuate throughout the GI tract for various reasons (e.g. fasting state). Recently, a similar study was published describing the use of encapsulated colicins for the eradication of *E. coli* in mice [36.] Colicins encapsulated into hydrogel particles were shown to be released from the protective coat at pH above 5 and reduce colonizing *E. coli* numbers in the gut and in feces, although complete eradication of the pathogen was not achieved [39]. Consequently, further research on klebicin formulation for the most efficient release in the lower intestinal tract is necessary. Importantly, new formulations for oral delivery, preferably using approved formulation agents such as Eudragit, should be studied in validated preclinical animal models to further optimize efficacy of bacteriocins as antibacterials for intestinal infections.

## Conclusions

This study demonstrated that successful colonization of the mouse intestinal tract by *K. quasipneumoniae* can be achieved but it requires the eradication of gut resident microbiota with an antibiotic. We also evaluated the antimicrobial activity of the orally delivered Eudragit S100-formulated klebicin in the mouse intestinal tract and show that thus formulated bacteriocins could be employed as oral antimicrobials for efficient control of antibiotic-resistant *Klebsiella*.

## Data Availability

All data generated during this study are included in this article.

## Abbreviations

PBS: Phosphate-buffered saline
GI: Gastrointestinal
*khe*: Haemolysin gene
HAI: Hospital-acquired infection
pen: Penicillin
strep: Streptomycin
met: Metronidazole
SGF: Sarcoma grow factor
SDS: Sodium dodecyl sulfate
MW: Molecular weight
DNA: Deoxyribonucleic acid
RNA: Ribonucleic acid
cDNA: Complementary Deoxyribonucleic acid
qRT-PCR: Qualitative real-time polymerase chain reaction
CFU: Colony-forming unit
